# Combining Chemoinformatics with Bioinformatics: *In Silico* Prediction of Bacterial Flavor-Forming Pathways by a Chemical Systems Biology Approach “Reverse Pathway Engineering”

**DOI:** 10.1371/journal.pone.0084769

**Published:** 2014-01-08

**Authors:** Mengjin Liu, Bruno Bienfait, Oliver Sacher, Johann Gasteiger, Roland J. Siezen, Arjen Nauta, Jan M. W. Geurts

**Affiliations:** 1 Department of Nutritional Sciences, FrieslandCampina, Amersfoort, The Netherlands; 2 Centre for Molecular and Biomolecular Informatics, Radboud University, Nijmegen, The Netherlands; 3 Molecular Networks GmbH, Erlangen, Germany; 4 Computer-Chemie-Centrum, University of Erlangen-Nürnberg, Erlangen, Germany; University of Houston, United States of America

## Abstract

The incompleteness of genome-scale metabolic models is a major bottleneck for systems biology approaches, which are based on large numbers of metabolites as identified and quantified by metabolomics. Many of the revealed secondary metabolites and/or their derivatives, such as flavor compounds, are non-essential in metabolism, and many of their synthesis pathways are unknown. In this study, we describe a novel approach, Reverse Pathway Engineering (RPE), which combines chemoinformatics and bioinformatics analyses, to predict the “missing links” between compounds of interest and their possible metabolic precursors by providing plausible chemical and/or enzymatic reactions. We demonstrate the added-value of the approach by using flavor-forming pathways in lactic acid bacteria (LAB) as an example. Established metabolic routes leading to the formation of flavor compounds from leucine were successfully replicated. Novel reactions involved in flavor formation, i.e. the conversion of alpha-hydroxy-isocaproate to 3-methylbutanoic acid and the synthesis of dimethyl sulfide, as well as the involved enzymes were successfully predicted. These new insights into the flavor-formation mechanisms in LAB can have a significant impact on improving the control of aroma formation in fermented food products. Since the input reaction databases and compounds are highly flexible, the RPE approach can be easily extended to a broad spectrum of applications, amongst others health/disease biomarker discovery as well as synthetic biology.

## Introduction

Chemical systems biology, a new discipline, linking chemical biology and systems biology, is currently drawing more and more attention [1]. One direction that has emerged is the development of novel strategies in which chemoinformatic and bioinformatic tools are integrated to interpret large high-throughput datasets.

A classical systems biology approach for analyzing cellular processes of microorganisms starts with the reconstruction of biochemical networks based on annotated genomes [2], [3]. However, those genome-scale metabolic models contain numerous gaps, only a small portion of which can be filled by computational methods or manual curation on the basis of known genetic/ biochemical datasets [4]. High-throughput analytical technologies for metabolomics, such as mass spectrometry (MS) and nuclear magnetic resonance spectroscopy (NMR), provide large data sets of chemical compounds from various biological systems [5]. Many of these compounds, particularly the products that are non-essential in metabolism, often cannot be mapped in the metabolic models due to the lack of biochemical and/or genetic information. In many cases, these secondary metabolites or their derivatives however can be of great economical value [6], [7] and/or possess potential health benefits [8]. Therefore, insight into their precursors and synthetic pathways could have a great impact with respect to the understanding and, ultimately, control of their production.

Flavor components in various fermented dairy foods are produced by lactic acid bacteria (LAB), which are present in starter cultures. Several reviews have summarized the flavor-forming pathways of LAB, especially the pathways originating from amino acids [9]–[12]. However, the flavor-forming networks are presumed to be more complex, and previous studies have only revealed small parts of this network. Thoroughly studied flavor-forming pathways still do not always meet the requirements to establish a comprehensive, curated predictive model for flavor formation. This often hampers the mapping of the revealed aroma compounds, for instance by gas chromatography-mass spectrometry (GC-MS), to a specific flavor-forming pathway. One of the main reasons for this is that although it is widely recognized that chemical modifications play an important role in flavor formation chemical reactions are usually not included in genome-scale metabolic models [2], [13].

In order to address the above mentioned knowledge gaps, we developed a novel approach, Reverse Pathway Engineering (RPE), which uses small molecules i.e. measured flavor compounds as input, and suggests the enzymatic or chemical reactions that can trace them back to known metabolic precursors. Thus, the production routes that synthesize the target compounds can be predicted.

The essential innovative step in the RPE approach is “Retrosynthesis”, an automatic process originally used by organic chemists to find methods to produce a given target compound by applying backward “retro reactions” to the target compound. This process was first posed by E.J. Corey and led to the development of the retrosynthesis program LHASA [14]. Many computer assisted programs for retrosynthesis have been developed since then [15]–[18]. However, a retrosynthesis program, which uses a database of known organic and biochemical reactions as a knowledge base, was not previously available.

In this paper, we present the novel RPE approach combining chemoinformatics (retrosynthesis) and bioinformatics (comparative genomics) to predict unrevealed reactions in metabolic pathways. We describe several cases, illustrating the strength of the approach to predict flavor-forming reactions/ pathways, especially those from leucine and methionine catabolism. Since the branched-chain amino acid degradation pathways, in particular the leucine degradation pathway, are relatively well studied [19]–[21], some routes from this pathway were used to validate the method. Novel reactions or alternative routes are proposed, such as the conversion of alpha-hydroxy-isocaproate to 3-methylbutanoic acid and the chemical reaction to form 2-methylpropanal from alpha-keto isocaproate. In addition, an enzymatic reaction to synthesize dimethyl sulfide from methanethiol in the methionine degradation pathway is proposed. Using bioinformatics analyses, plausible enzymes encoded in the genomes of LAB have been identified for catalyzing the predicted enzymatic reactions.

## Materials and Methods

### Flavor compounds

The flavor compounds used as inputs for the RPE approach were selected on the basis of literature and/or from GC-MS measurements [9]–[12], [22]. In this paper, the flavor compounds 3-methylbutanol, 3-methylbutanoic acid, 2-methylbutyric acid, 2-methylpropanal, and dimethyl sulfide were studied. The structures of the target compounds were obtained from the NCBI PubChem database (http://pubchem.ncbi.nlm.nih.gov/).

### Reaction database

The BioPath.Database [23] (Molecular Networks GmbH) is a manually curated biochemical reaction database, in which the reactions are stoichiometrically balanced, and where the reaction centers, as well as the bonds broken and formed during a reaction, have been marked (http://www.molecular-networks.com/databases/biopath.html). It contains reactions originally derived from the Roche Applied Science “Biochemical Pathways” wall chart and from a more extensive monograph [24]. In particular, for the examples of predicting flavor-forming pathways described in this paper, an extended version of the BioPath.Database has been used, with 438 additional reactions extracted from a *Lactobacillus plantarum* genome-scale metabolic model [25]. Moreover, 24 reactions were added from the flavor-forming pathways from sulfur-containing amino acids, including both enzymatic and chemical reactions [26]. After removing redundant reactions, the database contained 3,516 reactions in total, as the resource of reference reactions. The reactions of the BioPath.Database are annotated with a reversibility flag (reversible, irreversible, unknown) derived from the Roche Applied Science wall chart and primary literature. The additional 438 reactions of L. plantarum do not provide this information; four of the 24 reactions of the flavor-forming pathway were annotated with either reversible or irreversible.

### Prediction of flavor-forming reactions/pathways

In order to predict the unknown reactions or pathways leading to flavor formation, a software package called THERESA (THE REtroSynthetic Analyser) for designing organic synthesis reactions was used. THERESA derives a large portion of its knowledge from a reaction database. The extended BioPath.Database described above was utilized as the reaction pool. The combined system is called BioPath.Design as it allows the design of biotechnological processes. For each reaction of the BioPath.Database, BioPath.Design automatically extracted the reaction centers plus the transformation steps and then used them for RPE analyses. After the target structure was submitted, BioPath.Design searched its database of biotransformation rules for all reactions that could be applied to the target structure in the reverse direction (retrosynthesis). If a substructure match was identified, BioPath.Design could build a new complete synthesis reaction by adding the missing co-product(s), copying the atom-atom mapping numbers of the reaction centers to the target compound and generating the structure of the reactants. The predicted reactions were ranked based on the presence of reactants and predicted reactions in the database, as well as the simplicity of structures. The predicted reactions were manually inspected and a candidate reaction was selected based on the information of the corresponding reference reactions found in the database or on prior knowledge. When one of the plausible synthesis reactions was selected, the reactant could be used as the input compound to search for its precursor. This process was repeated iteratively until a known metabolic precursor was found and a complete synthetic route was retrieved. In order to consider the reversibility of biochemical reactions, the reversibility information stored with each reaction of BioPath.Database was used. In case of an irreversible reaction or if the reversibility information was unknown (especially those of the L. plantarum dataset), only one biotransformation rule was created, in case of a reversible reaction two biotransformation rules, one for each direction, were generated.

### Prediction of putative enzymes catalyzing the plausible reactions in LAB by comparative genomics

After a plausible reaction was proposed by BioPath.Design, a list of candidate enzymes which might catalyze the reaction was prepared on the basis of the reference reaction in the BioPath.Database or by inference from the transformation rule of the predicted reaction [27]. Detailed enzyme information, including literature references and sequence information when available, was obtained from the BRENDA database [28] by searching with the EC number or the enzyme name.

We used our previously described comparative genomics approach to predict putative enzymes for reactions [26]. In brief, sequences of the target enzymes, which had been characterized experimentally, were obtained from the UniProt/Swiss-Prot database (http://www.uniprot.org/). The genomes of the sequenced LAB and other microorganisms used in this study were obtained from the non-redundant genome databases, the NCBI microbial genome database (http://www.ncbi.nlm.nih.gov/genomes/lproks.cgi) and/or the ERGO database [29]. The retrieved protein sequence was used as a seed to perform a BLAST search against the microbial genomes to obtain their homologs. Multiple sequence alignments (MSAs) were created by MUSCLE [30], based on the homologous sequences of the target enzymes, as well as the sequences of the experimentally characterized enzymes from literature. Bootstrapped neighbor-joining phylogenetic trees were constructed within ClustalW [31] based on the MSAs and visualized by LOFT [32]. Orthologs of the putative enzymes in LAB genomes were proposed by phylogenetic analysis. The resulting enzymatic information was integrated into the retrosynthesized reactions and pathways for further analyses or applications. The outcome of a comparative genomics analysis was also used to verify the predicted reaction. For example, if no ortholog was found in the target organism, the selected reaction was regarded less likely to be correct.

### Reverse pathway engineering (RPE) approach

**Figure 1:**
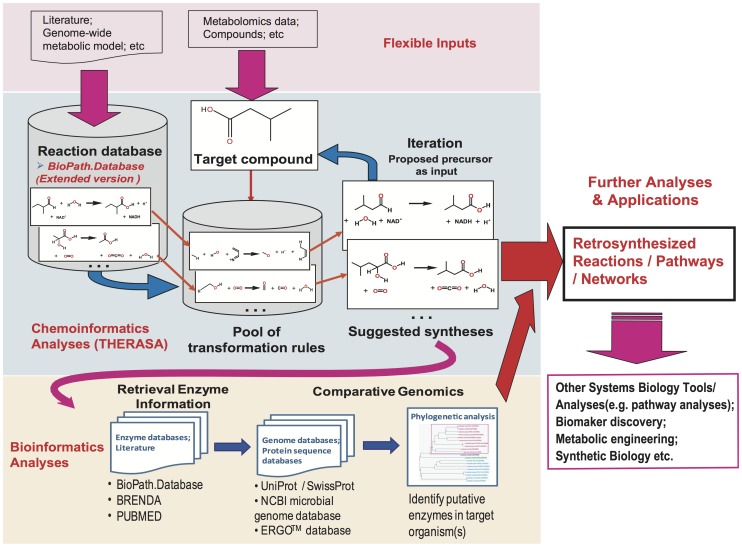
The Reverse Pathway Engineering (RPE) approach combines chemoinformatics and bioinformatics analyses. The approach enables a flexible input of target compounds and reaction databases, and can result in an output for various analyses or applications. Using 3-methylbutanoic acid as an input compound, two of the proposed synthetic reactions are shown as an example. The complete retrosynthesis trees can be found in Figure 4.

To close the gaps between the target chemical compounds and their metabolic precursors, we developed a novel approach named Reverse Pathway Engineering. The pipeline of the RPE approach, combining the scientific disciplines chemo- and bioinformatics, is shown in Figure 1. The chemoinformatics analyses are carried out by BioPath.Design and THERESA. Since the unrevealed reactions in metabolic pathways are usually not deposited in any biochemical or chemical database, a search will not retrieve the desired reaction. However, known enzymatic and chemical reactions can be used for generating a pool of transformation rules. BioPath.Design suggests synthesis reactions, including novel ones, by using the biotransformation rules based on those stored in a manually curated reaction database. Moreover, the reaction database of BioPath.Design can be adjusted according to specific tasks, in our case an extended biochemical reaction database named BioPath.Database (upper panel in Figure 1). The second step of the RPE approach is to apply bioinformatics analyses to predict the putative enzymes that can catalyze the suggested retrosynthesis reactions (lower panel in Figure 1). A list of candidate enzymes is proposed, based on either the reference reactions in BioPath.Database or by inferring them from the biotransformation rule of the predicted reaction. Since incorrect annotation of genes encoding enzymes in metabolic pathways is one of the causes of gaps in the reconstructed networks, a comparative genomics analysis is carried out to improve the annotations. Homologs in specific organisms are obtained from genome sequence databases by using the experimentally characterized protein sequences as the seed, after which orthologous genes are identified by phylogenetic analysis. The results of the bioinformatics analyses are used as an indication to verify the suggested reactions and, subsequently, to be integrated into the retrosynthesis routes.

## Results

### Validating the RPE approach: Mapping flavor molecules to the leucine degradation pathway

#### Leucine catabolism

**Figure 2:**
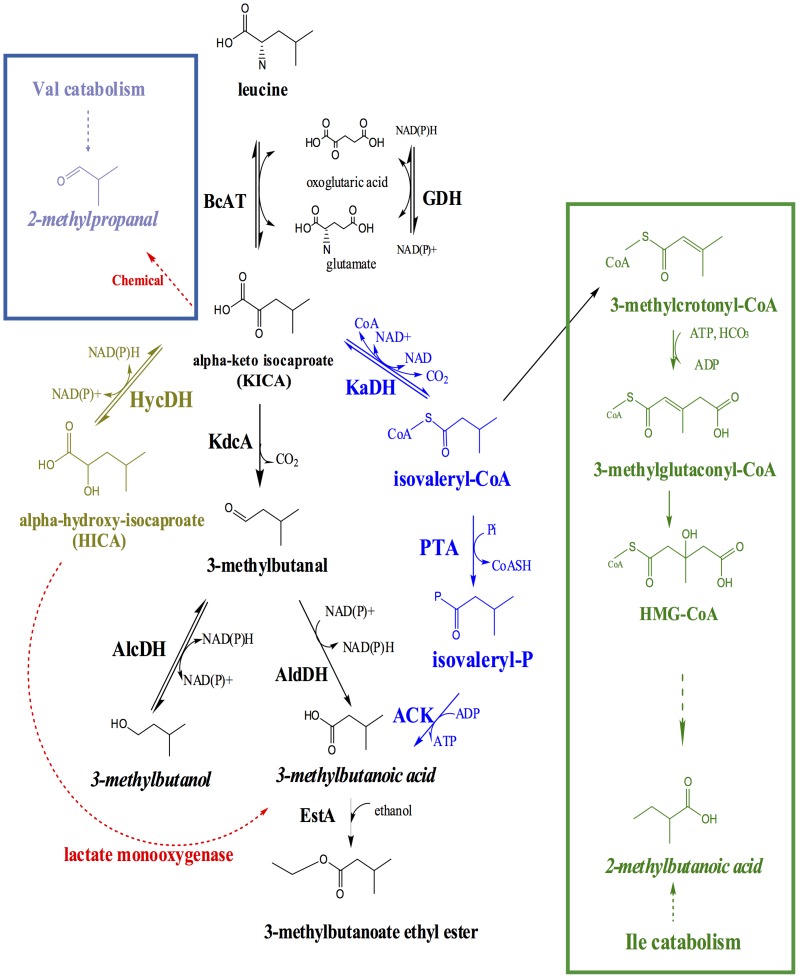
Leucine catabolism network in LAB, including the inter-conversion pathways between leucine degradation and valine catabolism (framed by blue box), and between leucine degradation and isoleucine catabolism (framed by green box). Three branches of the subsequent degradation of alpha-keto isocaproate (KICA) are indicated: i) conversion to the corresponding aldehyde, alcohol or carboxylic acid via alpha-keto acid decarboxylation (depicted in black) or ii) the oxidative decarboxylation (depicted in blue) or iii) an alternative route resulting in α-hydroxy-isocaproate (HICA), as depicted in gold. The flavor compounds used as input for RPE approach are indicated in italics. The novel predicted reactions are indicated by red dashed arrows. Enzymes names are: BcAT, branched-chain aminotransferase; GDH, glutamate dehydrogenase; HycDH, hydroxyacid dehydrogenase; KdcA, alpha-ketoacid decarboxylase; AlcDH, alcohol dehydrogenase; AldDH, aldehyde dehydrogenase; EstA, esterase A; KaDH, alpha-ketoacid dehydrogenase complex; PTA, phosphotransacylase; ACK, acyl kinase.

A leucine catabolism network was reconstructed on basis of several published studies [11], [20] (Figure 2). Some routes in this network were subsequently used to validate the RPE prediction method, on basis of selected flavor compounds.

Leucine catabolism can be divided into three major parts, as shown in Figure 2. The first part constitutes the main degradation pathway of leucine, initiated by an aminotransferase reaction converting leucine to alpha-keto isocaproate (KICA). The second part is the degradation of leucine to 2-methylbutanoic acid, as described by Ganesan et al. [20]. This pathway inter-connects the degradation of leucine and isoleucine since 2-methylbutyric acid is the corresponding carboxylic acid derived from isoleucine. The third part of leucine catabolism represents the inter-conversion pathway between leucine and valine.

The main branches of the leucine degradation pathway and the leucine and isoleucine inter-conversion route were used as positive controls for the validation of the RPE approach. The reactions highlighted in red are novel, so-far unrevealed reactions predicted by the RPE approach and will be described in detail in the following sections (Figure 2).

#### Main branches of the flavor-formation pathway from leucine

The main flavor products of leucine degradation are 3-methylbutanal, 3-methylbutanol, and 3-methylbutanoic acid, which provide cheesy, malty and sweaty odors, respectively [12]. Since 3-methylbutanal is one of the intermediates in the synthetic routes of the other two compounds, only 3-methylbutanol and 3-methylbutanoic acid were used as input for the retrosynthesis analysis.

**Figure 3:**
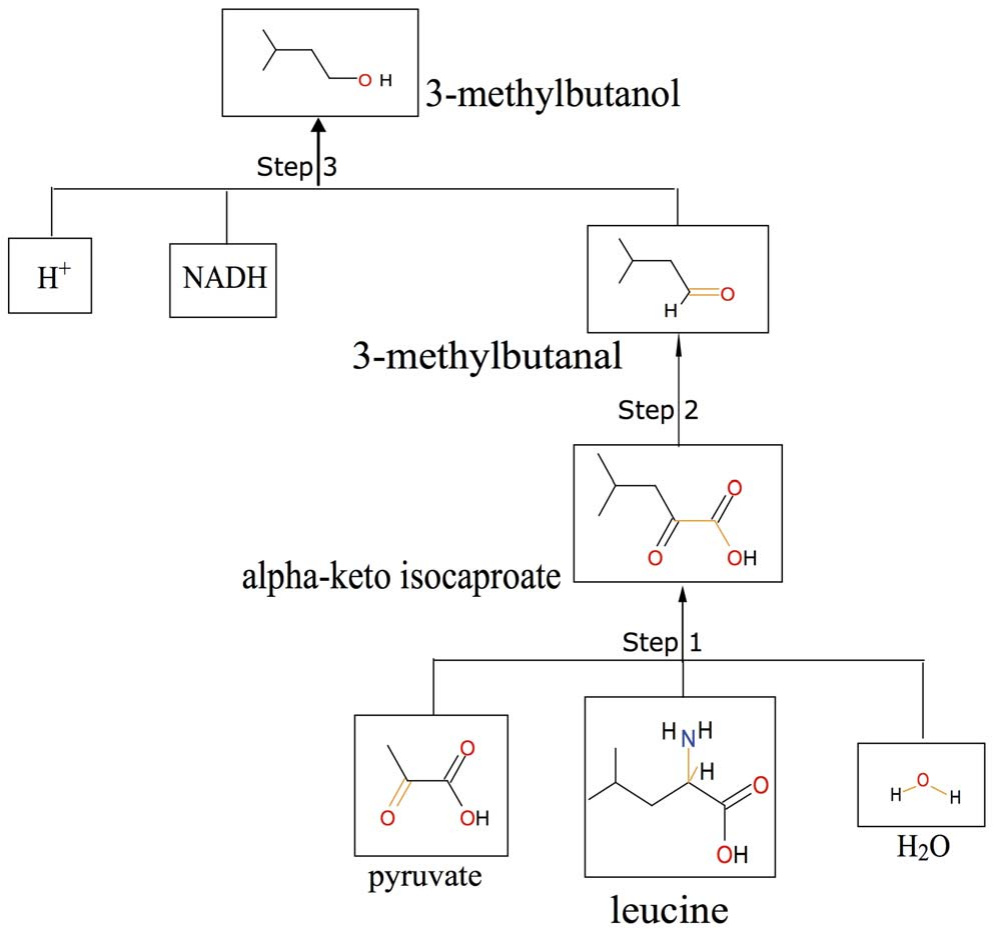
Leucine to 3-methylbutanol route as proposed by RPE. The retrosynthesis tree was obtained from BioPath.Design using 3-methylbutanol as input.

One of the retrieved synthetic routes of 3-methylbutanol is shown in Figure 3. First, leucine is converted to KICA by a transamination reaction, then further converted to 3-methylbutanal by a decarboxylation reaction. An alcohol dehydrogenase finally catalyzes the conversion of 3-methylbutanal to 3-methylbutanol with NADH as the cofactor. This 3-methylbutanol biosynthetic route, as predicted by RPE, is the same as the one described in literature (Figure 2). It should however be noted, that although at the transamination step (step1 in Figure 3) pyruvate is predicted to be the acceptor of the amino group based on its simpler structure, oxoglutarate was more often listed as the cofactor in the reference reactions. According to the frequency of the occurrences of the co-products in the reference reactions, oxoglutarate should be the cofactor for the predicted reaction, which is in line with experimental observation [33].

**Figure 4:**
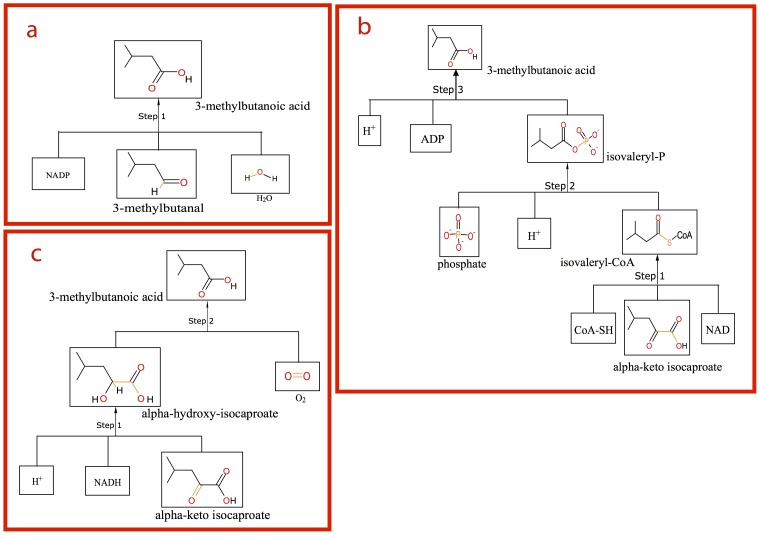
3-methylbutanoic acid synthesis routes from alpha-keto isocaproate as proposed by RPE. The retrosynthesis trees correspond to the pathways shown in Figure 2. (a) Proposed route for 3-methylbutanal to 3-methylbutanoic acid. (b) Proposed oxidative decarboxylation route converting alpha-keto isocaproate (KICA) into 3-methylbutanoic acid. (c) Proposed route converting KICA into 3-methylbutanoic acid via alpha-hydroxy-isocaproate (HICA). A novel reaction converting HICA into 3-methylbutanoic acid by lactate 2-monooxygenase was proposed (step 2).

When 3-methylbutanoic acid was used as input, three routes were predicted by the RPE approach (Figure 4). The first route is via oxidation of 3-methylbutanal (Figure 4a), similar to 3-methylbutanol biosynthesis described above. In the second route, 3-methylbutanoic acid is formed from KICA via isovaleryl-CoA and isovaleryl-phosphate catalyzed by a keto-acid dehydrogenase complex (Figure 4b). These RPE-proposed routes are the same as those described in literature (Figure 2) [34], [35]. The third one is a novel route (Figure 4c), and will be described in detail below (section “Leucine Degradation: Predicted novel reactions, Conversion of alpha-hydroxy-isocaproate to 3-methylbutanoic acid”).

#### Leucine-isoleucine inter-conversion pathway

The inter-conversion route between leucine and isoleucine degradation was also successfully predicted by the RPE approach. This inter-conversion route, in which isovaleryl-CoA is converted to 2-methylbutyric acid, has been proposed to occur in *Lactococcus lactis* during starvation as based on gene expression profiling, NMR, and Pathcomp analysis in KEGG [20] (Figure 2). By using 2-methylbutanoic acid as input and the intermediate compounds measured by NMR as selecting criteria, we could predict the synthetic route of hydroxymethylglutaryl-CoA from isovaleryl-CoA exactly the same as suggested by Ganesan et al., as well as the route of HMG-CoA to 2-methylbutanoic acid which is very similar as described in the study of Ganesan et al. (Figure S1) [20].

### Leucine degradation: Predicted novel reactions

#### Conversion of alpha-hydroxy-isocaproate to 3-methylbutanoic acid

Besides the two main biosynthesis routes of 3-methylbutanoic acid (i.e. via alpha-keto acid decarboxylation and oxidative decarboxylation) described above, a third route of 3-methylbutanoic acid synthesis from KICA via alpha-hydroxy-isocaproate (HICA) was proposed by RPE (Figure 2 and Figure 4c). Step 1 of this route was predicted to be a reduction reaction of KICA to HICA by hydroxy-acid dehydrogenase. This reaction has been described previously and a gene encoding the enzyme responsible for this conversion in *L. lactis* has been characterized [36].

**Figure 5:**
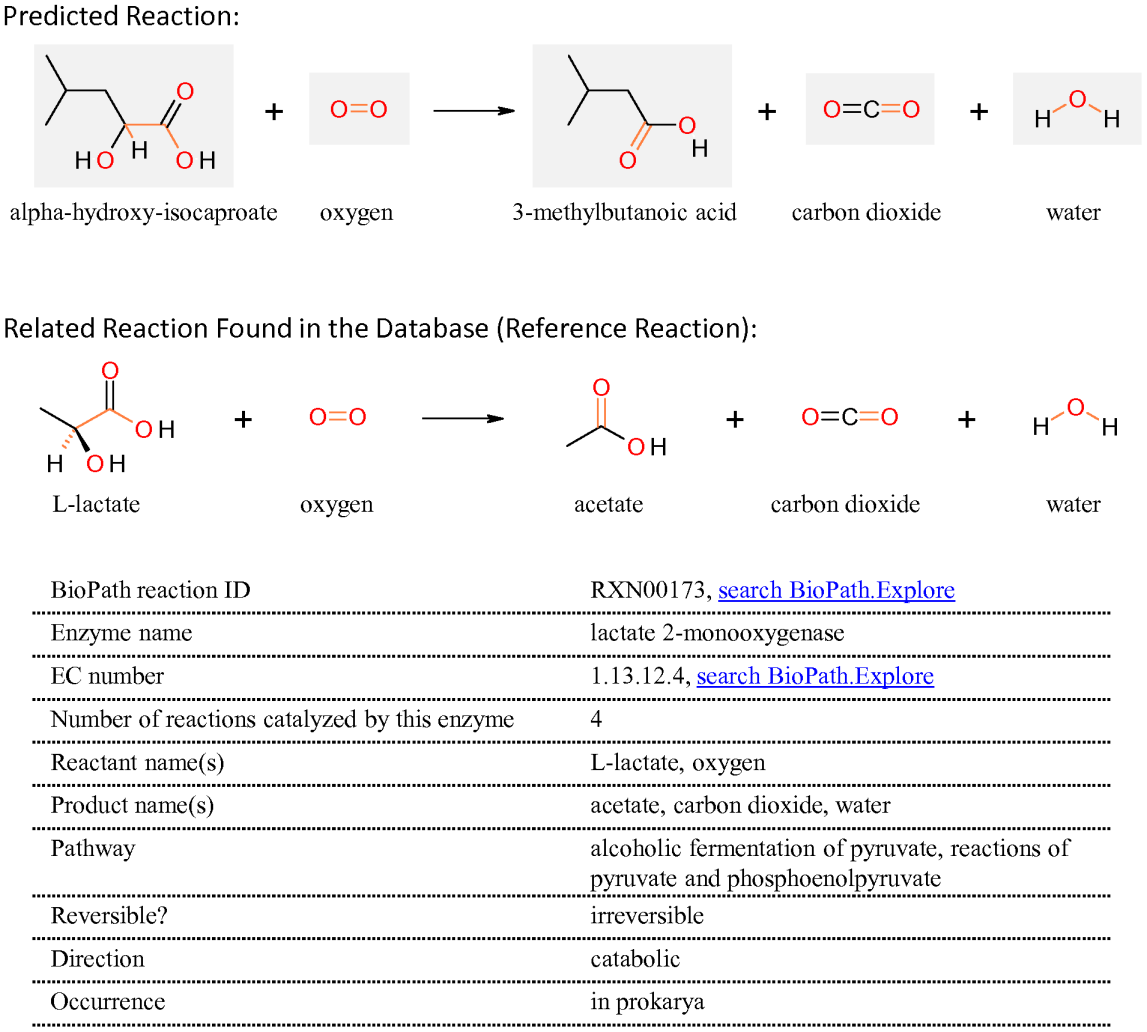
The suggested reaction converting alpha-hydroxy-isocaproate (HICA) to 3-methylbutanoic acid (adapted from BioPath.Design). The suggested reaction is shown in the upper part. One of the reference reactions is indicated together with the information on the enzyme which catalyzes it.

The second step of the predicted route suggests 3-methylbutanoic acid to be formed from HICA (Figure 4c and Figure 5). The lactate oxidation reaction catalyzed by lactate 2-monooxygenase is one of the reference reactions (Figure 5). As some enzymes have broader substrate specificities, and may catalyze analogous reactions, we hypothesize that lactate 2-monooxygenase can also catalyze the oxidation reaction of HICA.

**Figure 6:**
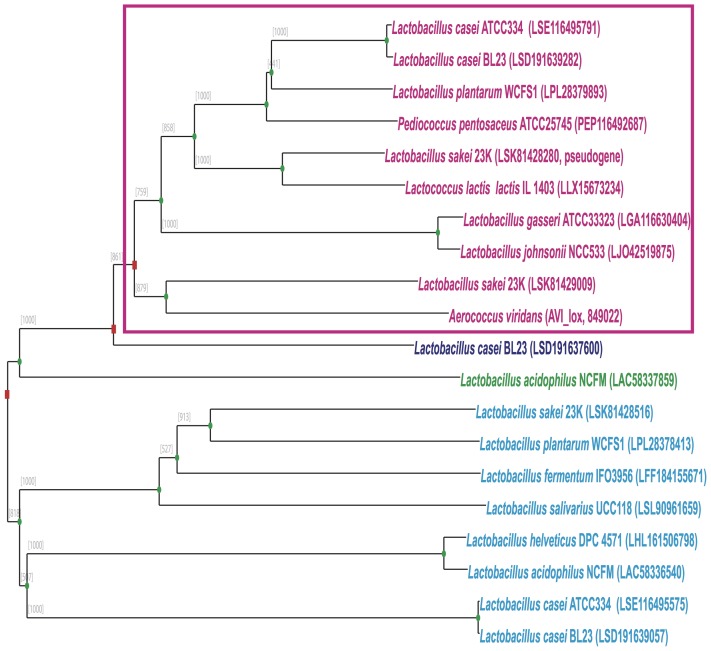
Bootstrapped (n = 1000) neighbor-joining tree of the LOX homologs from LAB. The functional equivalents (orthologs) of LOX from *Aerococcus viridans* are highlighted by the pink frame. Genome abbreviations and GI codes (NCBI accession codes) of the homologs are in parentheses. Different colors represent different orthologous groups. Diverse annotations have been assigned to the protein members in the lower groups, such as lactate dehydrogenase, isopentenyl pyrophosphate isomerase, and inosine-5-monophosphate dehydrogenase. Red squares indicate duplication events, and green dots represent speciation events.

In order to further substantiate this hypothesis, we performed additional bioinformatics studies. Lactate 2-monooxygenase activity was identified in *Aerococcus viridans*, *Mycobacterium smegmatis* and *Geotrichun candidum* [37]–[40]. It was found that this enzyme can utilize branched longer chain hydroxy acids as the substrate in *Mycobacterium smegmatis* [41], [42]. Using the protein sequence of the lactate 2-monooxygenase from *Mycobacterium smegmatis*, we identified its homologs in LAB by BLASTP. By performing comparative genomics analyses, the orthologs in LAB which are currently annotated as L-lactate oxidase (LOX) were identified (Figure 6). Interestingly, Yorita et al. found a mutant form of LOX from *Aerococcus viridians*, in which alanine 95 was replaced by glycine, yielding an enzyme isoform that has the same oxidase activity on L-lactate as the wild-type LOX, as well as an additional enhanced oxidase activity towards longer-chain hydroxy acids [42]. A multiple sequence alignment of LOXs shows that LOX of LAB also has an alanine conserved at the same position as Ala95 in LOX from *A. viridans* (Figure S2). This suggests that LOXs from LAB may only utilize lactate as a substrate, similar to the wild-type LOX from *A. viridans*. However, broader substrate specificity towards long-chain hydroxy acids could be obtained if the conserved alanine residue in LOX from LAB is mutated to glycine.

#### Chemical reaction converting alpha-keto isocaproate to 2-methylpropanal

In addition to the reactions catalyzed by enzymes, chemical conversions also play an important role in the process of flavor formation. For example, Bonnarme et al. [43] discovered that alpha-keto-γ-methylthiobutyrate (KMBA) can be chemically converted to methylsulfanyl-acetaldehyde (MTAC) and oxalic acid in the presence of Mn(II), alkaline pH and oxygen. MTAC is the precursor of various volatile sulfur compounds, such as methanethiol. This chemical reaction belongs to the reconstructed flavor-forming pathway from methionine that was included in the extended BioPath.Database in this study.

When 2-methylpropanal was used as the target compound for RPE, one of the reactions predicted by BioPath.Design was the decarboxylation of alpha-keto-isovaleric acid derived from valine, analogous to the decarboxylation reaction converting KICA to 3-methylbutanal described above. An exact match of this reaction was found by scanning the BioPath.Database, suggesting it has been stored in the database.

**Figure 7:**
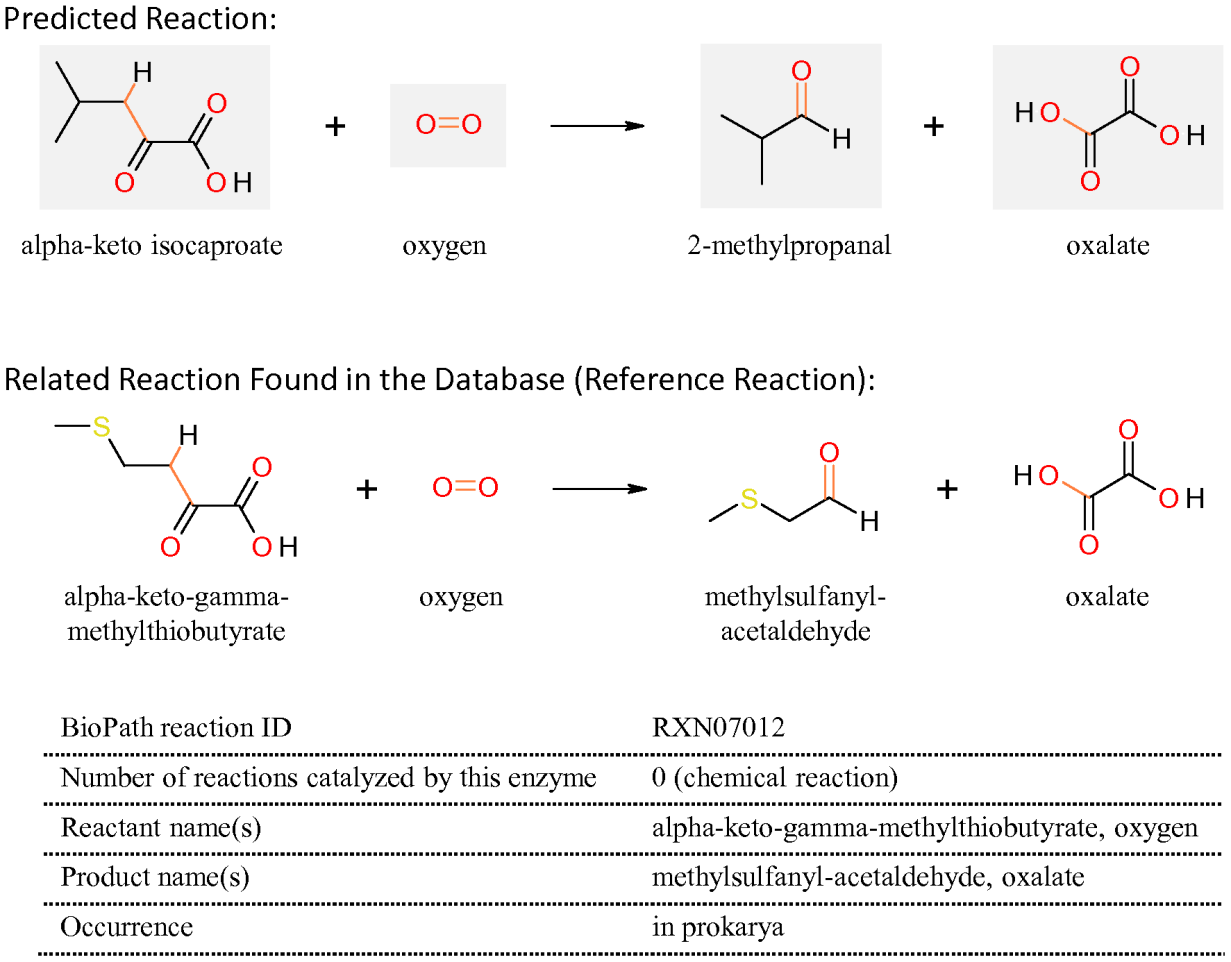
The predicted chemical reaction converts alpha-keto isocaproate (KICA) to 2-methylpropanal, connecting the leucine degradation and the valine degradation pathways. The reference reaction converting alpha-keto-γ-methylthiobutyrate to methylsulfanyl-acetaldehyde was derived from the additional part of the BioPath.Database containing the reactions of the flavor-forming pathways from sulfur-containing amino acid degradation.

A yet unrevealed novel chemical reaction converting KICA to 2-methylpropanal was also predicted, which inter-connects leucine and valine catabolism (Figure 2). The chemical conversion of KMBA to MTAC from methionine catabolism was proposed as the reference reaction (Figure 7). In the predicted reaction, KICA reacts with oxygen, resulting in 2-methylpropanal and oxalate. A literature survey confirmed that this chemical reaction indeed has been experimentally characterized by Smit et al. [21]. Interestingly, their study described that the chemical reaction can be modulated by Mn (II) concentration and oxygen as well as pH, a similar condition as was found for the reference reaction.

### Methionine degradation: predicted novel reactions

#### Enzymatic reaction converting methanethiol to DMS

Methionine catabolism is known as one of the main flavor-forming pathways, giving rise to various volatile sulfur compounds such as H_2_S, methanethiol, dimethyl sulfide (DMS), dimethyl disulfide (DMDS), and dimethyl trisulfide (DMTS) [26], [44].

Methanethiol, a compound derived from elimination of methionine catalyzed by a C-S lyase, is regarded as the precursor of DMS whose odor is described as “boiled cabbage, sulfurous” [45]. Two different hypotheses with respect to the reaction mechanisms of DMS formation from methanethiol have been proposed. One constitutes the chemical conversion of methanethiol to DMS [44], by either a methylation reaction or disproportionation reaction [46]. This chemical reaction, however, requires an extremely high temperature (350°C) and acid-base type catalysts [47], which are out of scope for fermentation reactions by LAB. The second hypothesis suggests that an enzymatic reaction is responsible for the conversion of methanethiol to DMS, utilizing S-adenosyl-L-methionine (S-AdoMet) as the methyl-group donor [48].

In order to predict the most plausible synthesis reaction, DMS was used as input for RPE. The reaction of methanethiol accepting a methyl group from S-AdoMet to form DMS and S-adenosyl-L-homocysteine (S-AdoHcy), in line with the second hypothesis, was predicted by BioPath.Design (Figure S3). The predicted reaction was based on a reference reaction which converts L-homocysteine into L-methionine using S-AdoMet as a methyl-donor. Several enzymes were proposed by the bioinformatics analyses as candidates for catalyzing this reaction, including homocysteine S-methyltransferase (EC 2.1.1.10), methionine synthase (EC 2.1.1.13) and thiol S-methyltransferase (TMT, EC 2.1.1.9). The first two enzymes are known for catalyzing the final step of methionine biosynthesis and are distributed widely among LAB [26], but their activity toward methanethiol is still unclear. TMT is mainly found in animals and plants, and has never been reported in LAB [49]. The genetically characterized TMT from *Brassica oleracea* (cabbage) [50] was used as “seed” to perform the comparative genomics analyses. TMT orthologs were identified in several *Streptococcus thermophilus* genomes and in the genome of *Propionibacterium freudenreichii* ATCC 6207 (Figure S4). The plausible TMT ortholog is in agreement with the observation that DMS is formed by *P. freudenreichii* in Swiss-type cheese [48], [51].

## Discussion

In the last decades, high-throughput analytical techniques, such as genomics, proteomics and metabolomics, have developed rapidly [5], [22], [52]. This increased analytical power urges for solutions in data storage, analysis and interpretation, especially with respect to secondary metabolites and their derivatives. Currently, many of the known flavor compounds cannot be traced back to their metabolic precursors, and it hampers the development of high-resolution flavor forming pathway representations. Here, we present a novel approach “Reverse Pathway Engineering”, which can be applied to retrieve the “missing links” in flavor-forming pathways of LAB, by combining chemoinformatics and bioinformatics analyses. To validate the RPE approach, the leucine degradation pathway was explored as it represents an important and thoroughly studied flavor-forming pathway in LAB [11], [20]. Even so, many of the described reactions from this pathway are still lacking in biochemical databases at this point in time, mainly because of the focus of these databases on primary metabolism. We were able to successfully replicate the main branches of the leucine degradation pathway and the inter-conversion route between leucine and isoleucine as described in literature with the RPE approach. After this successful validation of the approach we tested RPE for its capability to predict novel enzymatic reactions. This resulted in the prediction of several unrevealed reactions, such as the conversion of alpha-hydroxy-isocaproate to 3-methylbutanoic acid in the leucine degradation pathway and the formation of DMS from methanethiol in the methionine degradation pathway.

When our approach is compared to one of the previously published tools, Pathcomp [53], which has been used for the prediction of the inter-conversion pathway between leucine and isoleucine [20], RPE approach predicts a broader spectrum of reactions using the same dataset. As an example, RPE predicted that 2-methylacetoacetyl-CoA could be synthesized from either acetoacetyl-CoA or acetyl-CoA, rather than only from acetyl-CoA as reported by Ganesan et al. (Figure S1) [20]. This difference is probably due to the fact that the reaction databases for the pathway predictions significantly differ between both approaches. Pathcomp utilizes the KEGG database for predictions, while in our study the BioPath.Database was used. As Pathcomp is only able to retrieve known reactions stored in KEGG [53], unknown reactions cannot be predicted. In contrast, BioPath.Design is based on transformation rules, and can propose both known reactions as well as novel ones that were not originally stored in the database. In our validation experiment, on basis of the leucine degradation pathway, many reactions not originally stored in the reaction database were shown to be successfully predicted by RPE.

For the novel enzymatic reactions described in this study, the putative enzymes, as well as the candidate genes in LAB were proposed by bioinformatics analyses. The conflicting hypotheses on the synthesis of DMS from previous studies were elucidated in this study. In support of the hypothesis that DMS is formed enzymatically, our *in silico* analyses suggest both a plausible enzymatic reaction as well as the possible enzymes to catalyze it. The candidate reactions and enzymes can be used for directing future experiments to validate this hypothesis *in vivo*.

The predicted novel reactions not only increase the resolution of metabolic models, but also provide leads for metabolic engineering. Hydroxy acids such as HICA may have a negative effect on flavor formation since they share the same precursors as other flavor compounds such as 3-methylbutanol and 3-methylbutanoic acid [11], [26]. We propose an enzymatic reaction that converts the non-flavor compound HICA to the flavor compound 3-methylbutanoic acid. The homologs of the enzyme catalyzing this reaction were also found in several LAB genomes. As the study of *Aerococcus viridans* suggested that the homologous enzymes could gain the activity toward HICA by a site-directed mutation of the corresponding Ala95, the results of the RPE approach provide novel strategies for increasing the production of the flavor compound 3-methylbutanoic acid by utilizing HICA as a precursor. The novel enzymatic activities of LOX in LAB towards long-chain hydroxy acids can be introduced, similar to what has been described for the biosynthesis of shikimate pathway products by directed evolution of 2-keto-3-deoxy-6-phosphogalactonate aldolase in *E. coli* [54], or by adaptive evolution e.g. by stressing the bacterial cells with excess amounts of hydroxy acids.

The RPE approach is not limited to the prediction of enzymatic reactions, as chemical reactions are predicted as well. The predicted chemical reaction for forming 2-methylpropanal from KICA was proposed to take place under the same environmental conditions as the reference reaction which chemically converts KMBA to MTAC [21], [43]. This suggests that the experimental conditions of the corresponding reference reaction can be directly applied to the novel predicted chemical reaction, which may save time for exploring different conditions. In this way, RPE can play an instrumental role when applied to complex biological systems that use both enzymatic and chemical conversions.

To be noted, the reference reaction of any predicted chemical reaction should be present in the set of reconstructed flavor-forming pathways in the extended BioPath.Database. This shows the importance of compiling a comprehensive and suitable reaction database for the RPE approach. For this reason, an extended BioPath.Database was constructed with additional reactions from the genome-scale metabolic model of *L. plantarum* and from the reconstructed sulfur flavor-forming pathways. More enzymatic reactions from LAB metabolism and chemical reactions occurring during milk fermentation, which have been described in literature, can be added to this database for further database improvement [55]. The most time- and labor-consuming step of the RPE approach is the manual selection of the most likely reactions from a list of proposed reactions. Good ranking criteria for (semi-)automated selection procedures will be helpful for reducing this manual work. At the moment, our ranking criteria were based on combining the presence of reactants and predicted reactions in the database, as well as the simplicity of structures of the reactants. However, these criteria sometimes can cause errors, e.g. the prediction of a less likely cofactor as described in the results section. New criteria are being explored, also for different application cases, such as the degree of structural similarity between the compounds in the predicted reactions and the reference reactions, or the reaction conditions used for predicting chemical reactions.

The RPE approach uniquely connects chemical data all the way back to genomic data. This distinguishes it from other retrobiosynthesis methods such as ReBiT (Retro-Biosynthesis Tool) [56], which only covers part of the whole process, i.e. predicting enzymes in designed pathways. and Route Designer [15], which uses a set of retrosynthesis rules specific to organic chemistry, making it less suitable for biochemical questions.

The RPE approach can be extended to other application fields, thanks to the flexibility of using various reaction databases and target compounds. Possible future applications could be in the field of biomarker discovery. Recently, a metabolomics study described the correlation between human metabolic phenotypes and specific dietary preferences with respect to chocolate consumption [57]. In the study, it was demonstrated that several metabolic biomarkers could be connected to human host and gut microbial co-metabolism. Other studies have identified metabolites as biomarkers for the evaluation of disease risks [58], [59]. Our approach will be particularly helpful in tackling questions regarding the interplay of host-microbial co-metabolism by predicting the synthesis routes of measured metabolites, assigning plausible enzyme reactions and enzymes to mammalian cells and/or gut microbes. Leads for other application areas can also be expected, for instance in the field of synthetic biology, supporting the *in silico* design of biosynthesis pathways to construct microbial cell factories [60], [61].

## Supporting Information

Figure S1 **Proposed retrosynthesis routes of inter-conversion pathways between leucine and isoleucine degradation (green box in** **Figure 2** **).** The upper panel shows the biosynthesis routes to 2-methylbutanoic acid from acetoacetyl-CoA (upper left) or acetyl-CoA (upper right). The lower panel shows the biosynthesis route of HMG-CoA, the precursor of both acetoacetyl-CoA and acetyl-CoA, from isovaleryl-CoA. A previous study by Ganesan et al. only proposed the synthesis route via acetyl-CoA.
(TIFF)

Figure S2 **Multiple sequence alignment of the LOX orthologous group (pink group in** **Figure 6** **).** Residue Ala95 from LOX in Aerococcus viridans is indicated by a red arrow. This residue is conserved in all LAB orthologous sequences. A site-directed mutation of Ala95 to Gly converted LOX in Aerococcus viridans to a long-chain hydroxyacid oxidase.
(TIF)

Figure S3 **Predicted reaction which converts methanethiol to DMS using S-AdoMet as the methyl donor.** The reference reaction, methylation of L-homocysteine, is the final step of methionine biosynthesis.
(TIF)

Figure S4 **Phylogenetic tree of thiol S-methyltransferase (TMT) homologs from microorganisms by using the TMT protein sequence from Brassica oleracea as the seed.** The functional equivalents (orthologs) of TMT are highlighted by the red frame. Genome abbreviations and GI codes of the homologs are in parentheses. Different colors represent different orthologous groups. Diverse annotations have been found for the protein members in the other groups, such as methionyl-tRNA synthetase, hypothetical proteins.
(TIF)
