# Supplementary figures and images for: Combining Chemoinformatics with Bioinformatics: In Silico Prediction of Bacterial Flavor-Forming Pathways by a Chemical Systems Biology Approach “Reverse Pathway Engineering”
Source: PLoS One. 2014 Jan 8;9(1):e84769. doi: 10.1371/journal.pone.0084769 (PMC3885609; doi:10.1371/journal.pone.0084769)

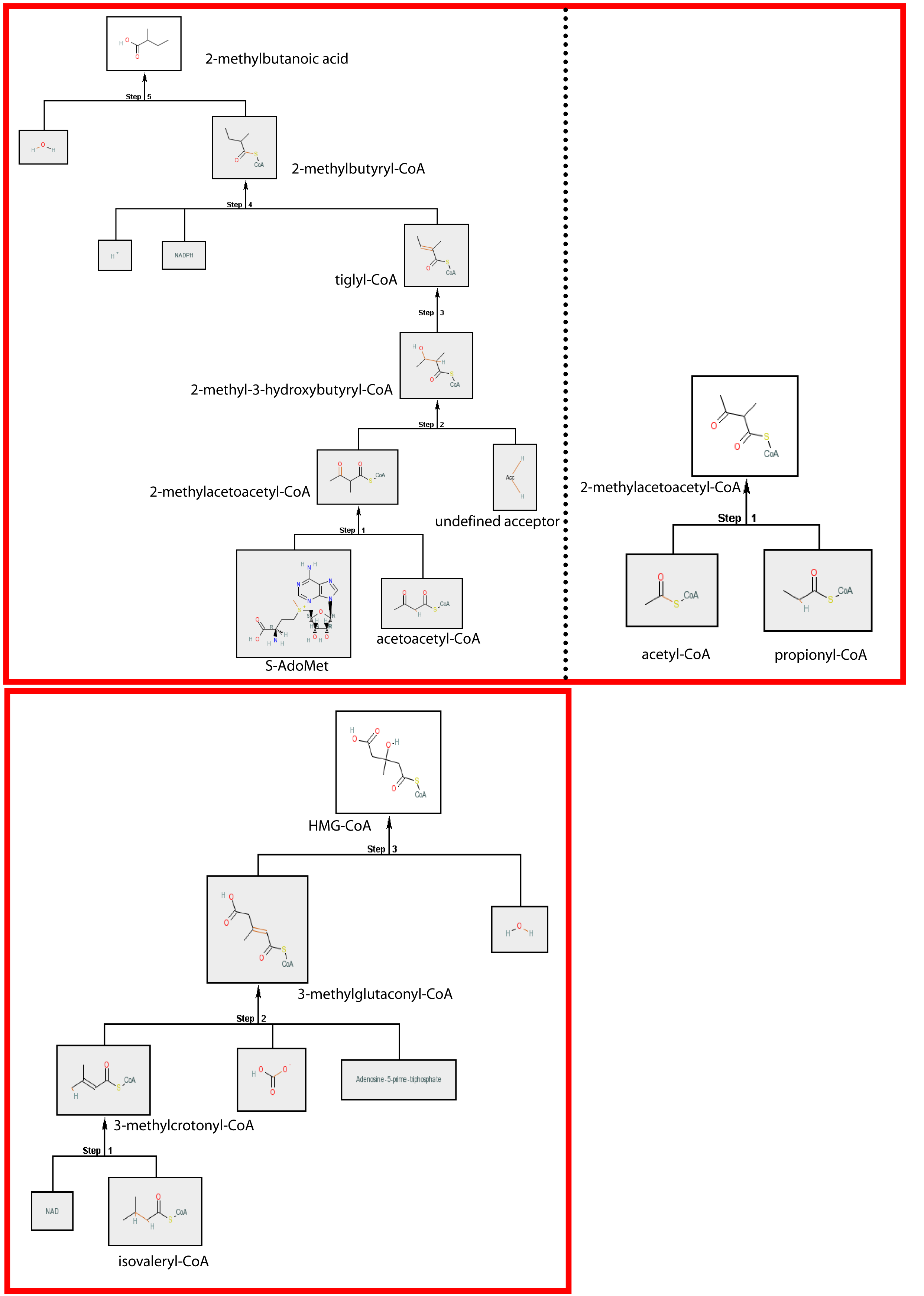

Supplement: Figure S1 — Proposed retrosynthesis routes of inter-conversion pathways between leucine and isoleucine degradation (green box in Figure 2 ). The upper panel shows the biosynthesis routes to 2-methylbutanoic acid from acetoacetyl-CoA (upper left) or acetyl-CoA (upper right). The lower panel shows the biosynthesis route of HMG-CoA, the precursor of both acetoacetyl-CoA and acetyl-CoA, from isovaleryl-CoA. A previous study by Ganesan et al. only proposed the synthesis route via acetyl-CoA. (TIFF) [file pone.0084769.s001.tiff]

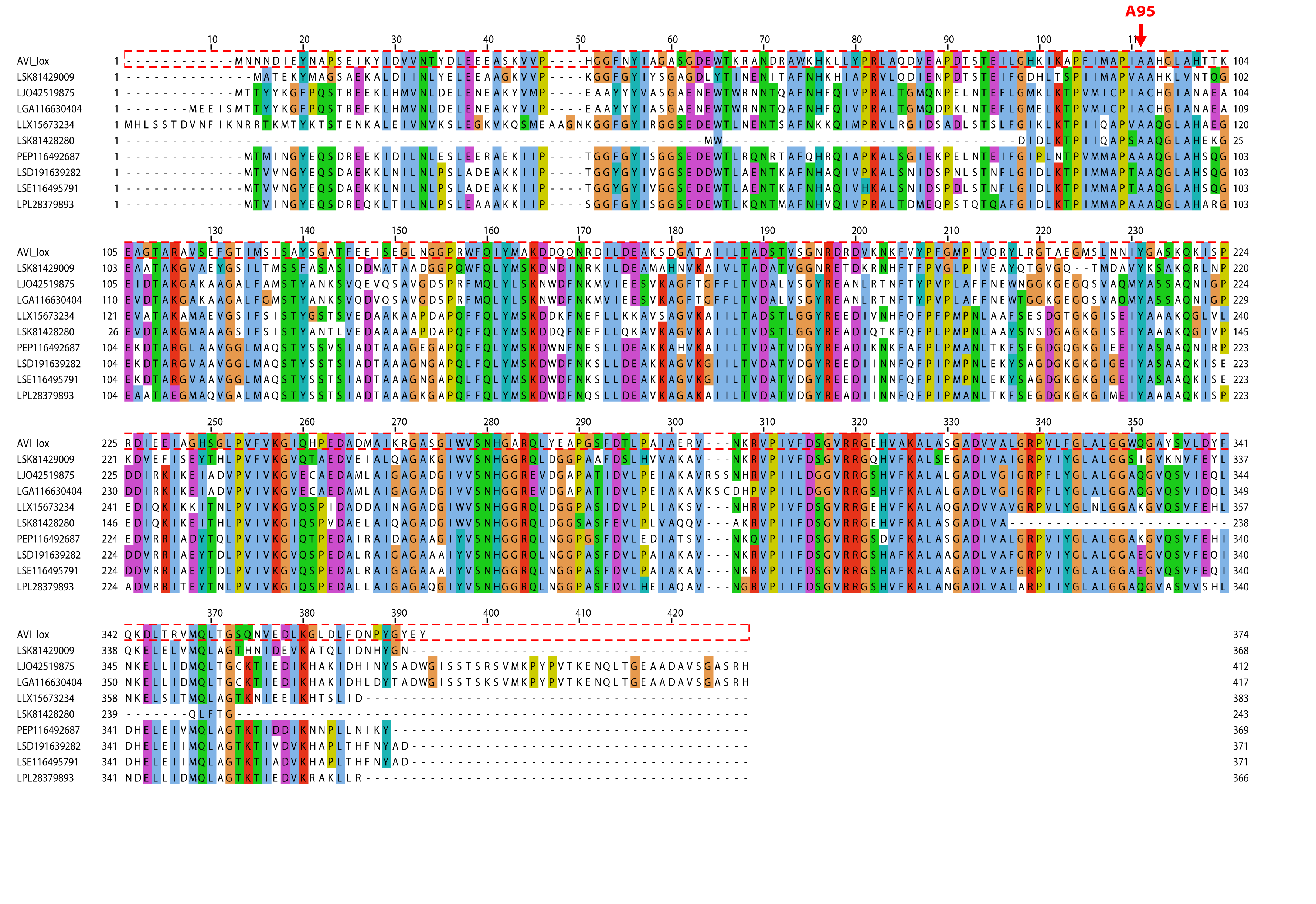

Supplement: Figure S2 — Multiple sequence alignment of the LOX orthologous group (pink group in Figure 6 ). Residue Ala95 from LOX in Aerococcus viridans is indicated by a red arrow. This residue is conserved in all LAB orthologous sequences. A site-directed mutation of Ala95 to Gly converted LOX in Aerococcus viridans to a long-chain hydroxyacid oxidase. (TIF) [file pone.0084769.s002.tif]

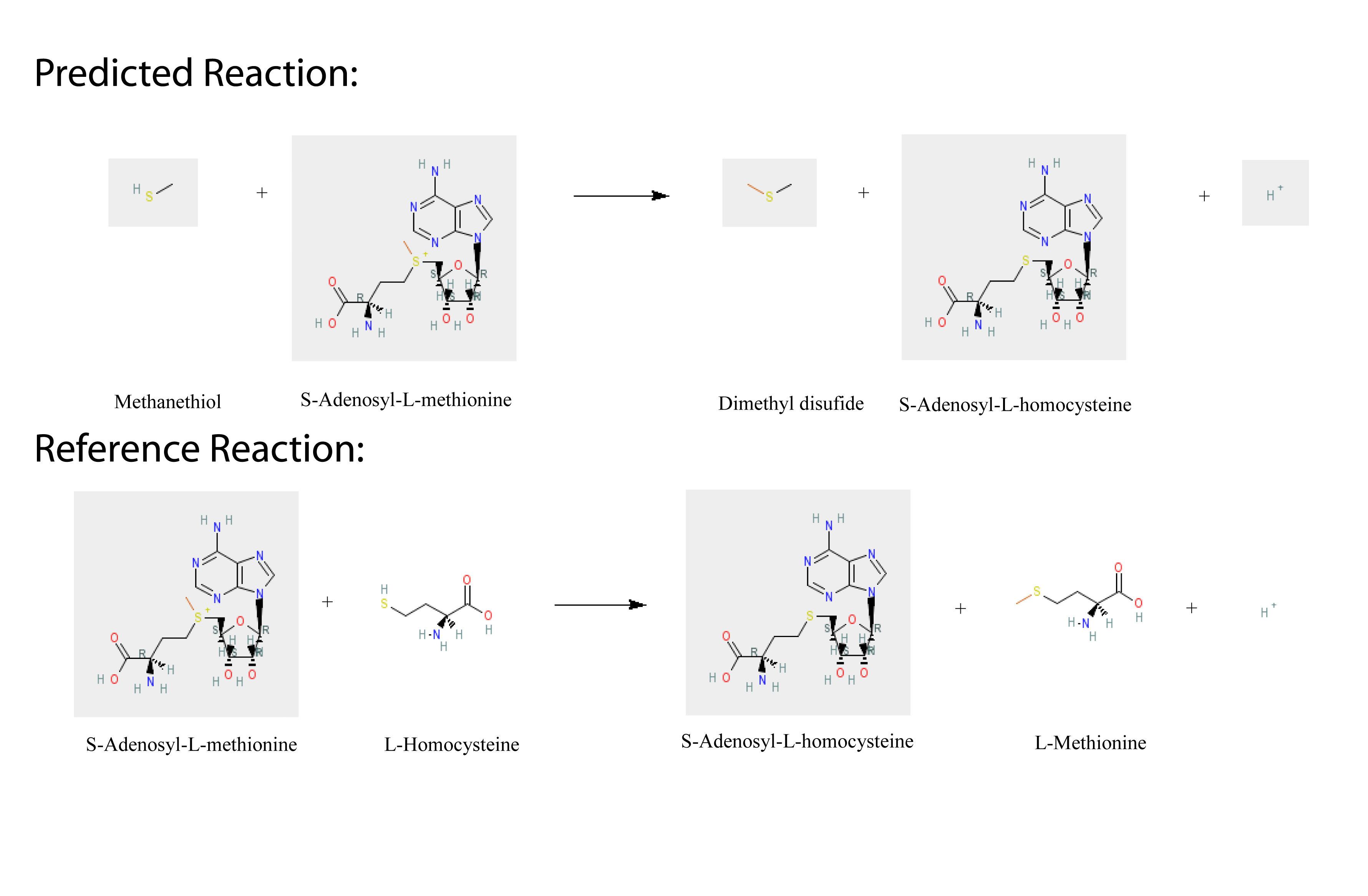

Supplement: Figure S3 — Predicted reaction which converts methanethiol to DMS using S-AdoMet as the methyl donor. The reference reaction, methylation of L-homocysteine, is the final step of methionine biosynthesis. (TIF) [file pone.0084769.s003.tif]

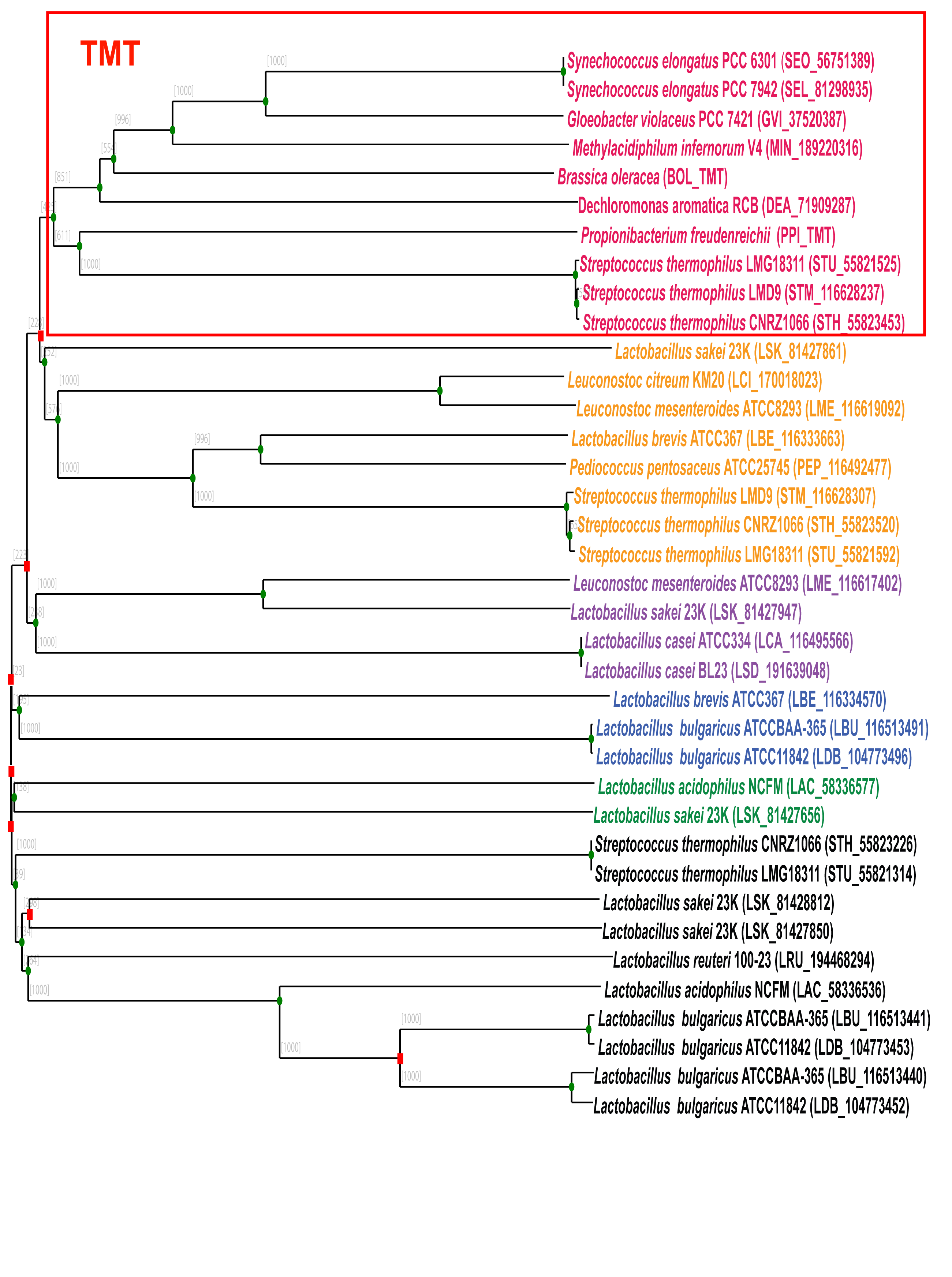

Supplement: Figure S4 — Phylogenetic tree of thiol S-methyltransferase (TMT) homologs from microorganisms by using the TMT protein sequence from Brassica oleracea as the seed. The functional equivalents (orthologs) of TMT are highlighted by the red frame. Genome abbreviations and GI codes of the homologs are in parentheses. Different colors represent different orthologous groups. Diverse annotations have been found for the protein members in the other groups, such as methionyl-tRNA synthetase, hypothetical proteins. (TIF) [file pone.0084769.s004.tif]
